# Prevalence of Depression and Depression Care for Populations Registered in Primary Care in Two Remote Cities in the Brazilian Amazon

**DOI:** 10.1371/journal.pone.0150046

**Published:** 2016-03-01

**Authors:** Edinilza Ribeiro dos Santos, Hsiang Huang, Paulo Rossi Menezes, Marcia Scazufca

**Affiliations:** 1 Department of Preventive Medicine, Faculty of Medicine, University of São Paulo, São Paulo, Brazil; 2 Health Sciences School, University of the State of Amazonas, Manaus, Brazil; 3 Department of Psychiatry, Cambridge Health Alliance, Harvard Medical School, Cambridge, United States of America; 4 Institute of Psychiatry and LIM-23, Clinics Hospital, Faculty of Medicine, University of São Paulo, São Paulo, Brazil; Mayo Clinic, UNITED STATES

## Abstract

**Background:**

The prevalence of depression has been widely studied in high-income countries and in large cities of low-income countries; however, little is known about the prevalence and treatment gap of depression in remote areas of the Amazonian region in Brazil.

**Objectives:**

The objectives of this study are to estimate the prevalence of depression in adults registered with the Family Health Strategy in two remote cities in the Brazilian Amazon and to investigate the proportion of individuals with depression that received mental health care.

**Methods:**

This is a cross-sectional study of an adult population registered with primary care clinics in the cities of Coari and Tefé, State of Amazon, Brazil. Depression was defined as a score of ≥10 on the Patient Health Questionnaire-9. Depression care was evaluated by asking participants with depression if they received antidepressants and/or had been seen by a health professional at a community mental health center in the three months prior to the interview. Poisson regression was used to examine the unadjusted and adjusted associations between depression and exposure variables.

**Results:**

The overall prevalence of depression was 19.1% (95% CI: 17.2–21.1), with 22.2% (95% CI: 19.3–25.0) among women and 16.0% (95% CI: 13.4–18.5) among men. The prevalence of depression in Coari and Tefé were 18.3% (CI 95% 15.7–21.0) and 19.9% (95% CI:17.2–22.7), respectively. Being a woman, lacking social support, increasing exposure to stressful life events and having a higher number medical comorbidities were consistently associated with depression. Lower educational attainment and income, tobacco use, and risky alcohol use were also associated with depression in the unadjusted analyses. Only 11.5% of those with depression were receiving antidepressants and/or visited the mental health care facility during the three months prior to the interview.

**Conclusion:**

Approximately one in five adults in our sample had depression. A high proportion of participants presented indicators of social disadvantage and other risk factors previously associated with depression worldwide. There was a large treatment gap for depression in the Amazonian region, which demonstrates the need for innovative models of depression care in primary care settings in Brazil

## Introduction

It is estimated that more than 350 million people in the world have depressive disorders [1]. Depression has been shown to be the second leading cause of years lived with disability in 2010 [2, 3]. This common mental disorder is associated with medical comorbidities [4, 5], suicide [6, 7], and increased health care utilization [8]. The treatment gap for depression is large [9], especially in primary care settings [10–14].

Although depression commonly occurs in primary care settings [15], various barriers exist to effective treatment of depression in primary care in low and middle income countries [16]. Although health systems have developed effective programs for chronic diseases such as hypertension and diabetes, infant-maternal care, and communicable diseases, systematic methods for detecting and treating depression remain suboptimal. Health care professionals are often overworked and do not have the time and the knowledge to diagnose and manage depression. Public stigma about depression also contributes to patients’ presentation of emotional complaints as somatic problems, which reduces the likelihood of correct diagnosis [9, 17].

Few studies have examined the prevalence of depression in the Brazilian population, and this information is even scarcer for the Amazonian region. A national study in Brazil shows a point prevalence of depressive symptoms of 28.3%, with the majority of cases (15.3%) presenting with severe depression. The Northern region presented the higher point prevalence of depressive symptoms than other Brazilian regions (37.3%) [18]. This study also showed higher prevalence of depressive symptoms among women, older adults, people with lower educational attainment and low income, and people who are single. Population based studies conducted in three cities in the Southwest and South regions of Brazil showed a prevalence of depression of 8.2% in Bambuí, 9.4% in São Paulo, and 20.4% in Pelotas [8, 19, 20]. Contrasting to the population based studies, the Brazilian National Household Survey [21] estimated that 7.6% of the adult population (~11.2 million people) received a diagnosis of depression. The disparity between the estimates showed by population based studies and the Brazilian National Household Survey are likely to reflect the treatment gap for depression in Brazil.

The aims of our study are to estimate the prevalence of depression in people registered with the Family Health Strategy (FHS) in two remote cities in the Brazilian Amazon and to investigate the the proportion of participants that visited the Community Mental Health Center in the three months prior to the interview and/or were currently receiving antidepressant medication depression care for those with depression. The FHS is the priority primary health care model of the Brazilian Unified Health System, and it is established all over the country [22]. The structure of the FHS is the same across the country, and consists of health teams with one family doctor, a nurse, a nursing assistant, and 7–12 community health workers. These health teams work in defined catchment areas with each team providing comprehensive and longitudinal care for up to 4,000 inhabitants. As of December 2014, there have been 39,228 teams implemented across the entire country, covering 59.0% of the Brazilian population [23].

## Materials and Methods

### Study design

This is a cross-sectional study of an adult population registered with primary care clinics with FHS teams in the cities of Coari end Tefé. These two cities are situated in municipalities with the same names in the central area of the State of Amazon, in Brazil. Data was collected between August 2013 and May 2014.

#### Study setting and sample

The State of Amazon is situated in the Northern area of Brazil and is divided into 62 municipalities, and borders three countries (Venezuela, Colombia and Peru) and five Brazilian States. The state has 3.5 million inhabitants with 52% living in the state capital, Manaus [24]. The State of Amazon has one of the smallest demographic densities (2.23 hab/Km^2^) among Brazilian states. Coari and Tefé municipalities are the fifth and sixth most populated municipalities in the State of Amazon, respectively. The river Solimões is the main access to these municipalities. The boat trip from Manaus, the capital of the Amazon State, to Coari and Tefé lasts at least 30 and 40 hours, respectively. The boat trip from Coari to Tefé lasts about 10 hours. These cities also have airports, but the main transport system for the population and goods are boats. The population of Coari municipality in 2010 was 75,965 inhabitants, and the population living in the city of Coari was 49,651 (25,911 aged 20 years or older) [25]. Tefé municipality had 61,453 inhabitants in 2010, of whom 50,069 lived in the city of Tefé (25,597 aged 20 years or older) [26]. The Human Development Index of the State of Amazon is 0.67 (medium development), and the Human Development Index of Coari and Tefé municipalities are 0.58 (low development) and 0.63 (medium development), respectively [27].

The sample size calculation for each town was based on an expected prevalence of depression of 20%, with a 95% confidence interval from 18% to 22%, and power of 80%. The sample in each town was calculated to be 818 participants. The expected prevalence of 20% was based on a previous population based study carried out in a city in the south of Brazil that used a similar methodology as in our study [20]. Eligible participants were all residents of the urban areas of Coari and Tefé cities, 20 years old or older, and registered in any of the primary care units with FHS of these cities. The sample was defined in two stages: identification of all eligible subjects and random selection of the study participants. The identification of eligible subjects in each Family Health Team (FHT) was based on the primary care unit registry. Name, date of birth, sex, and address of all eligible subjects were included in a database that was used to randomly select the study participants. The study participants were selected based on the sample size for each town (800 participants), the number of eligible subjects in each FHT, and the total number of eligible subjects in each town. For example, the sample size of a FHT with 1,846 eligible subjects in Coari (18,323 eligible subjects) was 81. We then calculated a proportional number of participants by sex in each age group (20–39, 40–59, > = 60) for each FHT, based on the information collected previously (age and sex of eligible subjects). Exclusion criteria included deafness, muteness, and difficulty understanding the questions asked by the interviewer. These individuals, along with those who declined to be interviewed or were not found by the interviewer, were replaced by individuals of the same sex and age group who were registered within the same FHT.

Ten trained interviewers carried out data collection. Selected subjects were visited at home and were invited to participate in the study. Interviewers received the names and addresses of subjects before visiting them at home. Up to three visits were carried out before the subjects was considered “not found” and replaced by another person. Interviews were carried out at participants’ homes after participants gave written informed consent. The interviewers filled out the questionnaires through the Open Data Kit Collect application 1.1.7. using mobile phones. This study was approved by the University of São Paulo Medical School Ethical Committee. Consent for the study was also obtained from the Health Secretariat of the municipalities of Coari and Tefé.

#### Measures

The outcomes investigated were prevalence of depression and depression care. Depression was defined as a score of ≥10 on the Patient Health Questionnaire-9 (PHQ-9) [28]. The PHQ-9 is based on the Diagnostic and Statistical Manual of Mental Disorders—Fourth Edition (DSM-IV), criteria for Major Depression Disorder [29]. The PHQ-9 diagnosis of major depressive disorder has been shown to be both valid and reliable compared to structured psychiatric interviews [30]. The answer to each item corresponds to how often depressive symptoms occur during the two weeks prior to the assessment. The Brazilian version of the PHQ-9 was used in our study [31]. Participants assessed as having depression (PHQ9≥10) were asked if they had been seen by a health professional (doctor, psychologist, or nurse) at the Community Mental Health Center in the three months prior to the interview (yes/no). Community Mental Health Centers are part of the Brazilian Universal Health System and provide treatments for mental disorders on an outpatient basis. The cities of Coari and Tefé have Community Mental Health Centers. Participants with depression were also asked if they were receiving medication for mental health problems (e.g. antidepressants) (yes/no). If they answered “yes,” they were then asked the name of the psychotropic medication. If the participant could not recall the name of the medication, he/she would be asked to show the research assistant the prescription for the medication. Medications were categorized as antidepressants, anxiolytics, neuroleptics, and mood stabilizers. The receipt of an antidepressant was considered a treatment for depression.

We used a standardized questionnaire to obtain information about exposure variables. We asked about gender, age (20–39, 40–59, > = 60 years old), skin color or race (mixed, white, and indigenous), marital status (married/civil union, single, widowed, separated), education (0–4, 5–8, 9–11, and > = 12 years of schooling) and personal monthly income (R$≤ 500, 501–1000, ≥1001). We also examined social support, stressful life events, smoking and alcohol habits, and medical comorbidities. The evaluation of social support asked about the frequency of meeting with relatives and friends/colleagues (at least once monthly or rarely/never). A list with ten stressful life events was used [32] and was scored based on the number of events participants had experienced in the past 12 months (0–1, 2, 3, > = 4). Smoking habits were assessed by asking participants if they currently smoked (Yes/No). Alcohol use during the last year was assessed with the Alcohol Use Disorders Identification Test–AUDIT [33] adapted and validated for Brazil [34]. Participants were classified as “no use and low risk use” and “risk use” (score ≥8). Self-reported morbidities were assessed by asking participants if they had any of the following chronic diseases: hypertension, diabetes, stroke, cancer, any lung related disease (asthma, bronchitis, emphysema), heart disease (myocardial infarction, angina, heart failure), liver problem (hepatitis, cirrhosis), digestive problems (stomach ulcer, gastritis, other intestinal problems), back or spinal column problems and arthritis or rheumatism. A variable with “number of morbidities” was used in the analyses (0, 1, 2, ≥3).

#### Analysis

Descriptive analyses were performed for the socio-demographic and clinical characteristics of participants (separated by cities). Overall prevalence of depression and the prevalence of depression for each city, with its 95% confidence interval (95% CI), were also calculated. Poisson regression was used to examine the crude and adjusted associations between depression and exposure variables. We constructed three analytical models. In the first model, city and socio-demographic factors were included; in the second model, social support and stressful life events were added to model 1; in the third (fully adjusted) model, tobacco use, alcohol use, and comorbid medical conditions were added to model 2. The third model shows which exposure variables are independently associated with depression. The likelihood ratio test (LRTEST) and the Wald were used to assess statistical significance. Prevalence ratio (PR) and 95% CI for the outcome depression are reported. Depression care was assessed by the proportion of participants that visited the Community Mental Health Center in the three months prior to the interview and/or were currently receiving antidepressant medication.

## Results

### Sample description

The lists from the FHS units yielded 34,838 eligible subjects (Coari = 18,323; Tefé = 16,515), and a random sample of 1,601 was then obtained. Seven hundred and twenty four participants had to be replaced primarily because of change of address (38.5%), address not found (17.0%), participant not in town for more than 30 days (15.7%), and refusals (15.5%). Only 14 subjects were replaced due to the exclusion criteria. Non-participation represented 31.2% of all individuals invited to take part in the study. One thousand six hundred and one individuals were included and interviewed (Coari = 805; Tefé = 826). Table 1 shows socio-demographic characteristics of the study sample, stratified by the two cities. Both cities have very similar characteristics, except for tobacco use (37.4% in Coari and 15.62% in Tefé). Among the entire sample, 12.0% (n = 195) had no income, while only 6.14% (n = 100) had individual monthly income greater than R$2,000.

**Table 1:** Socio-demographic and economic characteristics, social support, stressful life events, smoking habits, alcohol use, and physical morbidities.

Variables	Coari (n = 805)		Tefé (n = 826)	
	n	%	N	%
**Gender**				
**Male**	393	48.8	400	48.4
**Female**	412	51.2	426	51.6
**Age group**				
**20–39**	473	58.7	483	58.5
**40–59**	225	27.0	226	27.4
**60–94**	107	13.3	117	14.1
Ethnicity or skin Color*				
**Mixed and Black**	716	89.0	694	84.7
**White (including Asian)**	72	8.9	78	9.6
**Indigenous**	17	2.1	47	5.7
**Maritial status**				
**Married/civil union**	560	69.6	590	71.4
**Single**	163	20.2	165	19.0
**Widowed**	36	4.5	34	4.1
**Separated**	46	5.7	37	4.5
**Education (years of schooling)**				
**≥ 12**	175	21.7	181	21.9
**9–11**	204	25.3	221	26.8
**5–8**	170	21.2	173	20.9
**0–4**	256	31.8	251	30.4
Personal monthly income (R$)**^#^				
**≥1001.00**	138	17.2	143	17.5
**501.00–1000.00**	333	41.5	328	40.3
**≤ 500.00**	331	41.3	344	42.2
**Frequency of meeting with relatives**				
**At least once monthly**	728	90.4	715	86.5
**Rarely/never**	77	9.6	111	13.5
**Frequency of meeting with friends/colleagues**				
**At least once monthly**	641	79.6	628	76.0
**Rarely/never**	164	20.4	198	23.0
**Number of stressful life events**				
**0–1**	435	54.1	443	53.6
**2**	192	23.8	202	24.5
**3**	104	12.9	112	13.5
**≥ 4**	74	9.2	69	8.4
**Smoking habits (current use)**				
**No**	504	62.6	697	84.38
**Yes**	301	37.4	129	15.62
**Alcohol use**				
**No use or low risk use**	634	78.8	650	78.7
**Risk use**	171	21.2	176	21.3
Self-reported morbidities***				
**0**	275	34.3	270	34.0
**1**	218	27.2	231	29.1
**2**	177	22.0	165	20.8
**≥ 3**	132	16.5	127	16.1

*7 missing in Tefé.

**3 missing in Coari and 11 in Tefé.

^#^During the study period 1 US$ = 2.29 R$ (Brazilian Reals).

***4 missing in Coari and 37 in Tefé.

### The prevalence of depression and associated factors

The overall prevalence of depression was 19.1% (95% CI: 17.2–21.1), with 22.2% (95% CI: 19.3–25.0) among women and 16.0% (95% CI: 13.4–18.5) among men. The prevalence of depression in Coari and Tefé were 18.3% (95% CI: 15.7–21.0) and 19.9% (95% CI: 17.2–22.7), respectively.

Women, those with fewer years of formal education, lower individual income, less social support from family members and friends, more stressful life events, tobacco use, risky alcohol use, and more self-reported morbidities presented higher risk of having depression in the crude analyses (Table 2). Age, ethinicity/skin color, and marital status were not associated with prevalent depression. Women had higher risk of having depression than men, even in the fully adjusted model (model III). The association of prevalent depression with education and with personal income was slightly attenuated when all demographic and socio-economic variables were entered simultaneosly in the analysis (model I). These associations were markedly attenuated when the analyses were further adjusted by social support and stressful life events (model II). Controlling for life events and socio-demographic exposures led to a small decrease in the associations of prevalent depression and social support exposures (model II). The association of prevalent depression and life events was maintaned even after controlling for all exposures included in model III. Participants with four or more life events had approximately five times higher risk of having depression than those with none or only one life event. The association of prevalent depression and tobacco use lost its statistical significance when the analysis was controled for all exposures variables (model III), whereas the associations of prevalent depression with risky alcohol use and with increased number of self-reported morbidity were maintained (model III).

**Table 2:** Associations between prevalent depression with city of residence, socio-demographic characteristics, social support, stressful life events, smoking habits, alcohol use and physical morbidities (N = 1,572).

Variables	Crude analysis		Adjusted analyses					
			model I*		model II**		model III***	
	PR^¶^ (95% CI)	p^#^	PR(95% CI)	p^#^	PR(95% CI)	p^#^	PR(95% CI)	p^#^
**City**								
**Coari**	1	0.555	1	0.573	1	0.714	1	0.724
**Tefé**	1.06(0.86–1.30)		1.06(0.84–1.34)		1.04(0.82–1.31)		1.04(0.82–1.31)	
**Gender**								
**Male**	1	0.001	1	0.005	1	<0.001	1	<0.001
**Female**	1.40(1.14–1.73)		1.40(1.10–1.78)		1.50(1.17–1.92)		1.61(1.23–2.07)	
**Age group**								
**20–39**	1	0.747	1	0.129	1	0.564	1	0.114
**40–59**	1.01(0.80–1.27)		0.91(0.68–1.21)		0.98(0.73–1.29)		0.81(0.60–1.09)	
**≥ 60**	0.89(0.64–1.22)		0.63(0.40–0.99)		0.79(0.50–1.23)		0.62(0.39–0.99)	
**Ethnicity or skin Color**								
**Mixed and Black**	1	0.418	1	0.499	1	0.753	1	0.636
**White (including Asian)**	1.13(0.81–1.58)		1.14(0.78–1.66)		1.12(0.77–1.64)		1.20(0.82–1.75)	
**Indigenous**	1.30(0.82–2.05)		1.32(0.78–2.24)		1.14(0.67–1.93)		1.06(0.62–1.81)	
**Marital status**								
**Married/civil union**	1	0.364	1	0.394	1	0.706	1	0.760
**Single**	1.09(0.85–1.41)		1.17(0.87–1.58)		1.16(0.86–1.57)		1.13(0.84–1.53)	
**Widowed**	1.13(0.69–1.83)		1.19(0.65–2.17)		1.10(0.60–2.01)		1.07(0.58–1.96)	
**Separated**	1.40(0.94–1.83)		1.41(0.89–2.24)		1.19(0.75–1.91)		1.20(0.75–1.92)	
**Education (in years)**								
**≥ 12**	1	0.068	1	0.057	1	0.143	1	0.443
**9–11**	1.17(0.84–1.62)		1.13(0.78–1.63)		1.14(0.78–1.65)		1.13(0.77–1.64)	
**5–8**	1.31(0.94–1.83)		1.37(0.93–2.02)		1.20(0.81–1.77)		1.11(0.75–1.65)	
**0–4**	1.46(1.08–1.98)		1.64(1.11–2.42)		1.53(1.04–2.26)		1.35(0.91–2.00)	
**Personal monthly income (R$)**								
**≥1001.00**	1	0.006	1	0.093	1	0.199	1	0.320
**500.00–1000.00**	1.75(1.22–2.51)		1.53(1.02–2.29)		1.20(0.80–1.82)		1.16(0.77–1.74)	
**≤ 500.00**	1.73(1.21–2.48)		1.34(0.88–2.03)		0.96(0.62–1.47)		0.95(0.62–1.46)	
**Frequency of meeting with Relatives**								
**At least once monthly**	1	0.007			1	0.063	1	0.082
**Rarely/never**	1.44(1.10–1.88)				1.36(0.99–1.87)		1.34(0.97–1.84)	
**Frequency of meeting with friends/colleagues**								
**At least once monthly**	1	<0.001			1	<0.001	1	<0.001
**Rarely/never**	1.84(1.50–2.27)				1.61(1.26–2.08)		1.28(1.28–2.12)	
**Number of the stressful life events on the last 12 months**								
**0–1**	1	<0.001			1	<0.001	1	<0.001
**2**	1.89(1.43–2.50)				1.92(1.41–2.62)		1.78(1.30–2.43)	
**3**	2.80(2.11–3.74)				2.86(2.05–3.98)		2.58(1.84–3.60)	
**≥ 4**	4.90(3.81–6.31)				5.03(3.64–6.93)		4.08(2.90–5.63)	
** Smoking habits (current use)**								
**No**	1	0.015					1	0.468
**Yes**	1.35(1.05–1.72)						1.12(0.82–1.52)	
**Alcohol use**								
**No use or low risk use**	1	0.025					1	0.085
**Risk use**	1.29(1.03–1.63)						1.30(0.99–1.77)	
**Self-reported morbidities**								
**0**	1	<0.001					1	<0.001
**1**	1.16(0.85–1.58)						1.16(0.83–1.63)	
**2**	1.61(1.19–2.18)						1.39(0.98–1.96)	
**≥ 3**	2.63(1.99–3.46)						2.30(1.61–3.29)	

^¶^PR = Prevalence Ration

*Model I: Analyses adjusted for gender, age group, ethnicity or skin colour, marital status, years of schooling, income.

**Model II: Analyses adjusted for frequency of meeting with relatives, frequency of meeting with friends/colleagues, number of stressful life events, and all variables included on model I.

***Model III: Analyses adjusted for smoking habits, alcohol use, self-reported morbidities, and all variables included in models I and II.

^#^p-value: Wald test.

### Depression care

Table 3 shows the proportion of participants with depression receiving mental health care. Approximately 7% (n = 21) of these individuals were taking antidepressant, 8% (n = 25) visited a Community Mental Health Center in the past three months, and 11.5% (n = 36) received any mental health care (using antidepressants and/or visited the Community Mental Health Centre in the three months prior to the interview). Although the proportion of participants taking antidepressants in both cities was low, the proportion in Coari (9.5%) was more than twice that of Tefé (4.2%).

**Table 3:** Frequency of participants with depression receiving mental health care, by city and overall.

Depression Care	Overall (n = 313)	Coari (n = 148)	Tefé (n = 165)
**Currently receiving antidepressant**	21 (6.7%)	14 (9.5%)	7 (4.2%)
**Visited Community Mental Health Centre in past 3 months**	25 (8.0%)	12 (8.1%)	13 (7.9%)
Any depression care*	36 (11.5%)	19 (12.8%)	17 (10.3%)

*Currently receiving antidepressant and/or has visited Community Mental Health Centre

## Discussion

The results of this study show that prevalence of depression among the adult population registered with the Family Health Strategy in two remote cities in the state of Amazon was 19.1%. Being female, lacking social support, increasing exposure to stressful life events and having a higher number of self-reported morbidities were consistently associated with prevalent depression. Lower educational attainment and income, tobacco use, and risky alcohol use were also associated with prevalent depression in unadjusted analysis. Access to mental health care for those suffering from depression in these two cities was infrequent, as only 11.5% receiving antidepressants and/or visited the Community Mental Health Center in the three months prior to this study.

Although the Amazon region is globally perceived as a place of great biodiversity and natural resources, featuring tropical rainforest, beautiful rivers and a wide variety of cultures [35], there are important social and economic adversities the local population are confronted with on a daily basis. For example, the region has high rates of unemployment and poverty, a large population with low levels of literacy, and social isolation [36]. In line with the general picture of the region, a high proportion of the population of Coari and Tefé presented indicators of social disadvantage, contrasting with the ‘bucolic’ view of the Amazonian region, which characterizes the population as living in a beautiful landscape surrounded with an abundance of healthy food, without the usual stressors of urban areas associated with depression. In this context, the high prevalence of depression we found is not surprising. Our findings showed that the prevalence of depression in Coari and Tefé was above the prevalence of depression found in other Brazilian studies carried out outside of the State of Amazon [37]. Our results also indicated that the socio-demographic and economic risk factors associated with depression in the Amazonian region are similar to those found in other Brazilian regions and worldwide [38, 39].

As in previous studies, our results showed that prevalent depression was associated with increased number of stressful life events [40–42], increased self-reported comorbidities [4, 5, 43, 44] and lack of contact with family members [45–48]. Disruption of the hypothalamic-pituitary axis due to stressful life events has been postulated as a mediator for depression. Past studies have shown that chronic medical conditions are associated with a two to three times higher risk of having depression [49, 50]. Comorbid depression can also worsen medical outcomes because of poor adherence to self-care measures including medication adherence, diet, and exercise [51]. Although certain genetic factors may place individuals at higher risk for developing depression in adulthood [52], this risk may be partially lowered by modifying risk factors that are subject to change through social policies, such as life events and social isolation (e.g. traffic accident, physical violence, lack of employment).

We found very low treatment rates for depression in our sample of primary care patients. Low treatment rates in low and middle income countries have been attributed to scarcity of resources and a hesitation from the health care system to scale up interventions for depression [53]. One solution for improving depression care in the FHS may be to implement the collaborative care model, which has been shown to improve depression outcomes for depressed primary care patients [54]. Collaborative care employs behavioral care managers that use patient registries to keep track of a panel of patients with depression [55]. The behavioral care managers also deliver brief psychological interventions such as psychoeducation and problem solving therapy [56] and reviews cases with a psychiatric expert on a regular basis to provide stepped care recommendations for the primary care provider. Some of the risk factors we found for depression are modifiable and the intervention could potentially be implemented into the existing primary care platform of the FHS with either the nurse or nursing assistant taking on the role of the care manager. However, most collaborative care interventions have been tested in high income countries and no studies have been conducted in Brazil. Thus, effectiveness trials of this model of population based integrated mental health care need to be conducted in Brazil before widespread dissemination can be recommended.

Strengths of this study are the large sample size of primary care patients drawn from two cities in the Brazilian Amazon and the use of a structured assessment of depression used worldwide, which allows comparisons with results from other studies. Limitations include the fact that participants in this study are those who have registered in the FHS of urban areas of two middle-sized towns from the State of Amazon, therefore not representing the entire population of that region. However, many families in these two cities spend part of the year in rural areas of these municipalities working on agricultural activities, usually for their subsistence. Participation rates was another limitation. A major reason for non-participation was the frequency in change of address, which indicates that FHS records are not well updated. We did not validate the PHQ-9 for the population of the State of Amazon, but there are no reasons to suppose that they would behave in a different way from the Brazilian samples were the instrument was validated. Lastly, there is always a possibility of decreasing the actual prevalence of a disease when the number of people receiving treatment is not assessed. In the present study, we assessed depression care only among participants with depression which may have influenced the prevalence reported. However, the number of people receiving depression care was small in the group with depression, indicating that it is unlikely that the number of people receiving depression care among those without depression had a major impact on the prevalence reported.

The results of our study show that approximately one in five adults registered in the FHS in the cities of Coari and Tefé have depression. Furthermore, depression treatment rates are very low in this population. This large discrepancy between prevalence of depression and its treatment demonstrate the need for new models of depression care.

## Supporting Information

S1 Dataset Dataset of this study. (DTA)

## Abbreviations

FHS: Family Health Strategy
FHT: Family Health Team
PHQ-9: Patient Health Questionnaire-9
